# The Joint Effect of a Combination of Components From the Fruit of *Crataegus pinnatifida* Bge. Var. *major* N.E. Br. and the Root of *Salvia miltiorrhiza* Bge. With Exercises on Swimming in Focal Cerebral Infraction in Rat

**DOI:** 10.3389/fphys.2020.574535

**Published:** 2020-11-23

**Authors:** Shilan Ding, Wei Wang, Xiaojie Yin, Lan Wang, Leilei Gong, Fulong Liao, Rixin Liang

**Affiliations:** Institute of Chinese Material Medical, China Academy of Chinese Medical Sciences, Beijing, China

**Keywords:** *Crataegus pinnatifida* Bge. Var. *major* N.E. Br., *Salvia miltiorrhiza* Bge., swimming, focal cerebral infraction, shear stress, joint effect

## Abstract

**Background**: In our previous study, we found that the combination of a traditional Chinese medicine (TCM) and swimming could prevent atherosclerosis through a synergistic interaction. However, whether the combined application of active components from the fruit of *Crataegus pinnatifida* Bge. Var. *major* N.E. Br. and the root of *Salvia miltiorrhiza* Bge. (CPSM) and swimming has been effective in the prevention and treatment of focal cerebral infraction remained unclear. This work aimed to conduct detailed investigation on the joint effects of CPSM extract with swimming on focal cerebral infraction in rats and its underlying mechanisms.

**Method**: A photochemical method of the combination of Rose Bengal (RB) injection and cold-light source irradiation was performed to establish the rat focal cerebral thrombosis model. The pathological changes of the brain were observed by a DCP-7030 laser multifunction machine, and the protein levels of von Willebrand factor (vWF), vascular cell adhesion molecule-1 (VCAM-1), and intercellular adhesion molecule-1 (ICAM-1) were detected by Western blotting. Blood samples were collected to assay tissue plasminogen activator (t-PA), plasminogen activator inhibitor type-1 (PAI-1), endothelin-1 (ET-1), 6-keto-prostaglandin F1α (6-keto-PGF1α), and thromboxane B_2_ (TXB_2_). Finally, the gene expression of t-PA, PAI-1, and ICAM-1 in human umbilical vein endothelial cells (HUVECs) stimulated by tumor necrosis factor-α (TNF-α) was assayed *via* real-time (RT) quantitative PCR (qPCR).

**Results**: The joint effects of CPSM extract and swimming demonstrated significant interactions, which including increased blood perfusion, increased serum t-PA and 6-keto-PGF1α, decreased serum PAI-1 and TXB_2_, decreased protein levels of vWF, VCAM-1 and ICAM-1, and decreased ICAM-1 gene expression.

**Conclusion**: This research demonstrated that the combined therapy of CP and SM extracts with swimming could prevent focal cerebral infraction through interactions on the regulation of vascular endothelial functions and inflammatory factors. It stresses the promising effects of the drugs and shear stress of blood flow in prevention and treatment of thrombosis. The mechanism may be related to regulating the protein expression of vWF, VCAM-1, and ICAM-1, and downregulating the gene expression of ICAM-1.

## Introduction

Cerebral infraction commonly occurs because of arterial thrombosis, which results in occlusion in the blood vessel and generates hypoxia in the brain (Strong et al., 2007). At present, the cerebral vascular disease is characterized by high mortality rate and prevalence worldwide, including China (Carmichael, 2005). About 1.8 million neurons are lost every minute when appropriate treatment is not given (“time is brain”; Hu et al., 2019). Katrak et al. (2009) and Ikeda et al. (2013) have reported that the prognoses of patients with cerebral infarctions were poor with less efficacy of rehabilitation compared with those with cerebral hemorrhages. However, in the pathophysiology of infraction, hemodynamic shear stress, a pivotal factor, should be paid more attention (Tazraei et al., 2015). Several investigations have agreed that tissue lesions, such as thrombosis, are influenced by shear stress and alterations in blood flow within a stenosed artery (Wu et al., 1995). In recent decades, blood flow analysis in the cerebrovascular has become an important research topic in biomechanics. These blood vessels have complex geometry, including non-linear carves, constantly changing cross-sections, and several branches (Yan et al., 2020). A growing body of evidence has revealed that the impact of blood flow on endothelial cells (ECs) is primarily determined by two flow conditions: steady laminar flow and disturbed flow. The former is a protective factor, whereas the latter is an undesirable factor through thrombosis, inflammatory response, and endothelial dysfunction (Tanioka et al., 2019), indicating that disturbed flow plays an important role in the procession of cerebral infraction. At present, pharmacological therapy is the popular strategy for the prevention and treatment of cerebral infraction. However, whether cerebral infraction can be intervened by no pharmacological methods is less studied. The World Health Organization recommends the practice of regular exercises to prevent and ameliorate cerebrovascular diseases. A growing number of clinical and basic research data show that physical exercise is crucial in inhibiting thrombosis (Stralen et al., 2008).

The innermost layers of all blood vessels, ECs, are constantly exposed to forces. For example, mechanical forces in the form of shear stress generated by blood flow (Xu, 2020), and hemodynamic forces modulating endothelium functions by the expression endothelial secretion associated genes. Gong et al. (2018) claimed that fluid shear stress may stimulate the expression of tissue plasminogen activator (t-PA). Also, it may reduce the secretion of plasminogen activator inhibitor type-1 (PAI-1; Gong et al., 2018). Von Willebrand factor (vWF) is a critical component of the hemostatic system. The basal secretion of vWF from ECs is the principal determinant of individual’s baseline plasma vWF levels, whereas endothelial vWF release can also be induced by several biochemical agonists and biomechanical forces such as increased shear stress (Choi and Lillicrap, 2020). Based on previous studies, vWF, as a biological marker for arterial and thrombotic diseases has involvement with focal cerebral infraction. Endothelin-1 (ET-1) is a highly potent vasoconstrictor with considerable efficacy in numerous vascular beds. As reported recently, ET-1 can modulate organ blood flow with exercise under specific conditions (Rapoport and Merkus, 2017). The antithrombotic effect is evaluated by prostacyclin (measured as 6-keto-prostaglandin F1α, 6-keto-PGF1α) and thromboxane (measured as thromboxane B_2_, TXB_2_; Cui et al., 2018), which are closely associated with shear stress. Otherwise, cerebral infraction manifests as an inflammation state of the vascular wall. The changes that functionally promote lesion formation at these susceptible sites are EC membrane aberrant regulation of vascular cell adhesion molecule-1 (VCAM-1) and intercellular adhesion molecule-1 (ICAM-1; Qian et al., 2002).

Management of cerebral infraction can be achieved by using antithrombotic agents, such as antiplatelet drugs. However, most antithrombotic agents, when administered systemically, can induce bleeding at locations far from the site of action (Qian et al., 2002). A clinical study has shown that outpatient’s long-term use of aspirin in combination with other antiplatelet drugs increases the risk of bleeding, which results in cerebral hemorrhages (Bhatt et al., 2006). Searching for other effective agents and investigating their modes of action are important to select better candidates for future clinical application and to increase our understanding of the molecular mechanisms underlying arterial thrombosis *in vivo*. In the recent years, emphasis in research was given to alternative traditional Chinese medicine (TCM) for the management of cerebral infraction/ischemia (Zhao et al., 2017). Many drugs of TCM are being conducted in the prevention and treatment of cardiovascular diseases in China for thousands of years. Study on promoting blood circulation and removing blood stasis is the active field of research of the integration of traditional and western medicine in China (Liu et al., 2012). *Crataegus pinnatifida* Bge. Var. *major* N.E. Br. (CP) and *Salvia miltiorrhiza* Bge. (SM) are a typical herbal synergic pair in TCM. Various reports on the biological activities and blood concentrations of CP and SM components, such as proanthocyanidin B_2_, salvianolic acid B, and tanshinone II_A_, have found (Zhang et al., 2005; Zuo et al., 2006; Gong et al., 2018). Our previous study showed that the combination of a TCM and swimming may prevent and treat vascular diseases through a synergistic interaction that promotes thrombolysis, blood circulation, and vascular endothelium function (Wang et al., 2015). In screening and analyzing potentially active components of this herbal pair, we found that the combination of effective components (proanthocyanidin B_2_, salvianolic acid B, and tanshinone II_A_) from the fruit of CP and the root of SM has pharmacological activity. Zhang et al. (2013) have found that the effective components of CPSM are water-soluble extracts of CP, water-soluble extracts of SM, and fat-soluble extracts of CP. The optima ratio of each component was as follows: 84, 128, and 25 mg kg^−1^.

The present study aims to explore the possible synergistic effect and underlying mechanisms of the joint effects of CPSM extract with swimming on rats of cerebral infraction.

## Materials and Methods

### Reagent and Materials

Rose Bengal (RB) Na salt was purchased from Beijing coolaber Technology Co., Ltd. (China). Evans blue wau8s obtained from Beijing solarbio Technology Co., Ltd. (China). Primary polyclonal antibodies against ICAM-1 and VCAM-1 were from Absin Bioscience Inc. (Shanghai, China). vWF polyclonal primary antibody was purchased from Proteintech Group, Inc. (Chicago, United States). β-actin polyclonal primary antibody was from Beijing biosynthesis biotechnology Co., Ltd. (China). Goat anti-rabbit horseradish peroxidase (HRP) was acquired from Applygen Technologies Inc. (Beijing, China). Human umbilical vein ECs (HUVECs) were presented by Cui Hongyu, who works at Institute of Chinese Material Medical, China Academy of Chinese Medical Sciences. TNF-α protein was from PeproTech Inc. (Rocky Hill, United States). All kits for RNA isolation, cDNA synthesis, and real-time (RT) quantitative PCR (RT-qPCR) detection were purchased from Beijing TIANGEN Biotech Co., Ltd. (China).

### Experimental Animals’ Preparation

Healthy male Sprague-Dawley (SD) rats, weighing 210–230 g with a grade of specific-pathogen-free, were bought from SPF (Beijing) Biotechnology Co., Ltd. (SCXK 2014–0004; Beijing, China). The rats were randomly divided into 10 groups, including one sham-operated group (*n* = 6) and nine treatment groups (*n* = 6 each). The rats were maintained under standard laboratory conditions, given free access to sterilized food and purified water for 3 days *ad libitum* before the experiment, temperature 25 ± 1°C, relative humidity 40%, and a 12 h light/dark cycle. All animal experiments were approved by the Laboratory Animal Ethics Committee of the Institute of Chinese Material Medical, China Academy of Chinese Medical Sciences (Beijing, China). All animal studies were carried out in accordance with the guidelines and regulations for the care and use of laboratory animal of Center for Laboratory Animal Care, China Academy of Chinese Medical Science.

### CPSM Extract Formulation

CPSM extract were consisted of aqueous extract of CP fruit (CPA), aqueous extract of SM (SMA), and liposoluble extract of SM (SML). Almost, all the compounds were extracted from SM and CP fruit by the Chemistry Department of Institute of Chinese Materia Medica, according to the previously developed method by the author of Zhang et al. (2013). It was also proved that the main component of SM aqueous extract was salvianolic acid B; the main component of SM liposoluble extract was tanshinone IIA; and the main component of CPA was proanthocyanidin B_2_ by high-performance liquid chromatography (HPLC) analysis. Dosage trial of a combination of SMA (84 mg kg^−1^), CPA (128 mg kg^−1^), and SML (25 mg kg^−1^) exerts better antithrombotic effect; therefore, CPSM extract was prepared as a mixture at the ratio of 128: 84: 25 of CPA: SMA: SML.

### Drug Administration

The rats in the treatment groups were treated with CPSM extract at the doses of 237 and 474 mg kg^−1^ body weight by gavage administration for 2 weeks. The rats in the sham-operated group received the same amount of pure water by oral administration.

### Experimental Design

For this experiment, a fractional factorial design was employed to examine the two factors, swimming and CPSM extract administration, in rats with focal cerebral ischemia. The three levels of each factor were as listed below: swimming (no swimming, 20, and 40 min per day) and the dosages of CPSM extract (no CPSM, 237, and 474 mg kg^−1^ in rat per day). Nine groups of combined treatment were included in the 3 × 3 factorial design. CPSM extracts were given to the rats at the dosages of 0, 237, or 474 mg kg^−1^ by gavage daily, 6 days per week. The amount of swimming was selected according to our previous studies (You et al., 2011) with minor modifications, 20 min training protocol was considered as training of medium-strength, and 40 min training protocol was considered as high-strength training. Rats were placed in a plastic swimming pool (100 cm × 60 cm × 80 cm) containing water (35 ± 1°C, depth of 60 cm). The exercise duration was gradually increased by 10 min per day until each subsequent day until the maximum swimming duration of 20 or 40 min was reached (Kaufman et al., 1994), 6 days per week. During the implementation stage of the swimming protocol, we supervised every swimming session to ensure that every rat was exercising. The swim training continued until the end of the second week.

### Photochemically Induced Focal Cerebral Thrombosis in Rats

Cerebral thrombosis was induced photochemically on the basis of the procedure modified by Shi et al. (2012). After 2 weeks of intervention, all rats were anesthetized using 10% chloral hydrate at a dose of 0.3 g kg^−1^ *via* intraperitoneal injection. In brief, anesthetized rats were placed in a stereotactic frame, and body temperature was maintained at 36.5–37.5°C using an electrical heating pad. A small incision was made in the skin over the right femoral vein, and RB was injected *via* a catheter. At the dorsal part of the head, the skull was exposed by a median incision of the skin. The periost was gently removed, and the right parietal bone was identified. Then, the cranial window with a diameter of 6 mm was opened with a center of 3 mm on the right side of the sagittal seam and 3 mm behind the coronal seam using a micro-sculpture grinding machine. The cerebral blood flow in the area of the cranial window was detected for 5 min by laser speckle imaging (Perimed, Sweden). A fiber optic bundle of a cold light source was centered on the brains of the cranial window using a micromanipulator at 2.5 mm posteriorly and 2.5 mm laterally. The brains were then illuminated through the intact skull for 15 min starting 5 min after the intraperitoneal injection of RB with the dosage of 125 mg kg^−1^, and the intensity was 3,000 K. The sham-operated control group was treated similarly; however, the animals were not illuminated after receiving RB. Afterward, the blood flow of each group in the cranial window was detected for 5 min again by laser speckle imaging. After 1 h, all the animals were sacrificed.

### Pathological Assessments

After the surgical procedure, the brains of the rats were removed, the infarction area and vascular injury area were measured by DCP-7030 laser multifunction machine (Brother, China). The infarction area and vascular injury area were analyzed by the automated image analysis software (Image J, United States National Institutes of Health, Bethesda, MD, United States). The results are expressed as the percentage of cerebral infarction area to the area of the right hemisphere, and the percentage of the area of vascular injury to the area of the right hemisphere, respectively.

### Determination of Serum t-PA, PAI-1, ET-1, 6-keto-PGF1α, and TXB_2_

When the surgical procedure of all rats was over, blood samples were collected from femoral artery. About 5 ml, whole blood sample was collected and then centrifuged at 3,000 rpm for 15 min at 4°C to determine the contents of t-PA, PAI-1, ET-1, 6-keto-PGF1α, and TXB_2_ (chemically stable hydration product of TXA_2_ which is cleared rapidly from the circulation). t-PA, PAI-1, ET-1, 6-keto-PGF1α, and TXB_2_ levels in serum were measured by radioimmunoassay kit (Shanghai Xinfan Biotechnology Co., Ltd., China).

### Western Blotting Assay of vWF, VCAM-1, and ICAM-1

After the surgical procedure, the brains of the rats were removed. Western blotting analysis was performed as described previously (Yu et al., 2014). The infarcted brain tissues were lysed in high efficiency RIPA cold lysis buffer at a weight-to-volume ratio of 1:4. The homogenate was centrifuged at 12,000 rpm for 20 min at 4°C, and the protein concentration was assayed using the bicinchoninic acid (BCA) protein assay (Pierce, Rockford, United States). Equal amounts of total protein from each sample were loaded in each lane and separated using sodium dodecyl sulfate polyacrylamide gel electrophoresis. Proteins from gels were transferred onto a nitrocellulose filter membrane (Bio-Rad, United States). The membranes were washed, blocked, and incubated with rabbit anti-ICAM-1 polyclonal antibody (1:2,000 dilution), rabbit anti-VCAM-1 polyclonal antibody (1:1,000 dilution), rabbit anti-vWF polyclonal antibody (1:1,000 dilution), rabbit anti-β-actin antibody (1:10,000 dilution), and then with HRP-conjugated secondary antibody diluted at 1:1,000. The bands were revealed using enhanced chemiluminescence detection reagents (Pierce, Rockford, United States). The intensities of the protein bands on the image were quantified using the image J software.

### Cell Culture of HUVECs

Primary cultures of HUVECs were cultured in 25-cm^2^ flasks coated with gelatin in modified Roswell Park Memorial Institute (RPMI)-1640 medium supplemented with 10% fetal bovine serum (FBS; Gibco Life Technologies, United States) and other growth factors as reported in the literature (Abe et al., 2011; Rochier et al., 2011). All cell cultures were kept in a humidified incubator at 37°C with 5% CO_2_, and the media was refreshed every 3 days. Cells from passages 3 to 7 were used for the subsequent experiments.

### Mechanical Stress Exposure

When cultures were 80–90% confluent, trypsin ethylenediaminetetraacetic acid (0.25%, Gibco Life Technologies, United States) was added to detach them from the flasks, and HUVECs were seeded on fibronection (Fn, Solarbio, China) coated glass slides at a density of 2–3 × 10^7^ cells ml^−1^. The cells were exposed to shear stress, 0.1 Pa (low shear stress, 1 dyn/cm) and 0.9 Pa (9 dyn/cm, physiologically relevant fluid shear stress conditions) in the presence of various CPSM extract concentrations (0, 150, and 300 μg ml^−1^) for 12 h, utilizing the BioFlux 1000 (Fluxion Biosciences, United States). The flow of the perfusion medium was regulated by a computer-controlled pump, which can be programmed to generate different flow profiles. Then, 60 ng ml^−1^ TNF-α was added to each well and incubated for 2 h.

### Determination of Gene Expression by RT-qPCR

Total RNA of the HUVECs was extracted using the RNA simple Total RNA Kit according to the manufacturer’s instructions. Total RNA samples were purified using an RNA clean kit. The RNA concentration and purity were determined using a Biospec-nano spectrophotometer (Shimadzu Corporation, Japan). Then, the total RNA was reverse-transcribed using the FastKing RT Kit. RT-qPCR was performed to verify t-PA, PAI-1, and ICAM-1 genes with the reference gene β-actin. Primer pairs were designed *in silico* based on selected sequences by using Primer Premier 5.0 software (Premier Biosoft, United States). Sequences of primers and probes are as follows: β-actin forward primer 5'-CGGGACCTGACAGACTACCTC-3', and reverse primer 5'-GTACTCCTGCTTGCTGATCCA-3'; PAI-1 forward primer 5'-CTGGGTGAAGACACACACAAAAG-3' and reverse primer 5'-CACAGAGACAGTGCTGCCGT-3'; t-PA forward primer 5'-ATGCCCGATTCAGAAGAGGA-3' and reverse primer 5'-GTTGAAACACCTTGGCTCGC-3'; and ICAM-1 forward primer 5'-ATCTGTGTCCCCCTCAAAAGTC-3' and reverse primer 5'-CCATCAGGGCAGTTTGAATAGC-3'. All selected primers were custom-ordered from a commercial supplier (Beijing RuiBiotech Co., Ltd., China). RT-qPCR was performed in 96-well plates with an ABI 7500 Real-Time PCR System (Applied Biosystems, United States) by using a Talent qPCR PreMix (SYBR Green). Each reaction mix went through the following conditions: 95°C for 3 min; 40 cycles of 95°C for 3 s, 60°C for 30 s. For the data of relative gene expression obtained from the 7500 System Software, the 2^-ΔΔCt^ method was applied in analysis.

### Statistical Analysis

The measurement data conform to the normal distribution. The homogeneity of variance was tested by Levene’s statistics. Statistical analysis in this report was performed with SPSS version 19.0 (SPSS, Chicago, IL, United States). All experimental data are expressed as the mean ± SDs. Comparisons of factor levels between the sham-operated group and model group were analyzed by using the independent-sample *t*-test. The general liner models (GLM) univariate procedure in SPSS was performed for two-way ANOVA to test for synergism, and a Bonferroni *post hoc* test was used to show the differences between each pair of factor levels. A value of *p* < 0.05 was considered statistically significant.

## Results

### Pathological Assessment of Response to Induce Focal Cerebral Ischemia in Rats

#### Description of Successful Modeling

**Figure 1:**
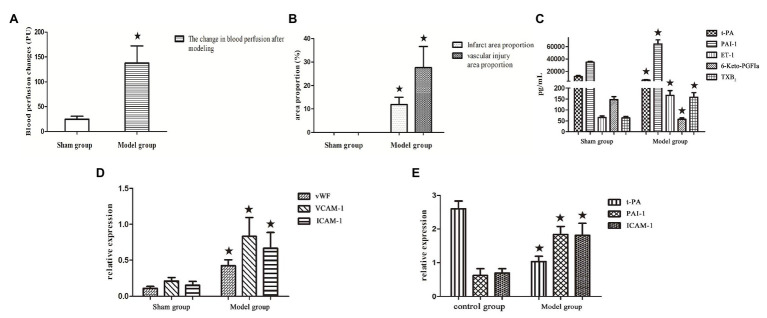
**(A–D)** Bar graphs of blood perfusion change, cerebral infarction area, vascular injury area, and endothelial factors. Protein expression in the model group and sham-operated group. **(E)** Bar graphs of gene expression in the model group and control group without the intervention of shear stress and drugs. All data are expressed as the mean ± SD. Comparisons between the sham group and model group were analyzed using the independent-sample *t*-test. A value of *p* < 0.05 was considered statistically significant.

The success of the experimental model is mainly illustrated by the following two indicators:

1. The difference of the change in blood perfusion signal irradiated by cold light source using laser Doppler flowmetry before and after modeling was evident. The changes in blood perfusion in the model group were −137.72 ± 34.39 PU, whereas in the sham-operated group, the changes were −4.65 ± 5.87 PU. As shown in Figure 1A.
2. The sham-operated group showed no evident histopathological changes. Compared with the sham-operated group, a large dyed red area, and Evans blue-stained vascular injury area of focal cerebral ischemia in the model group showed hemorrhagic infarction and developed thrombosis (Figure 1B), indicating that the preparation of this animal model was successful.

#### The Combination of CPSM Extract and Swimming

**Figure 2:**
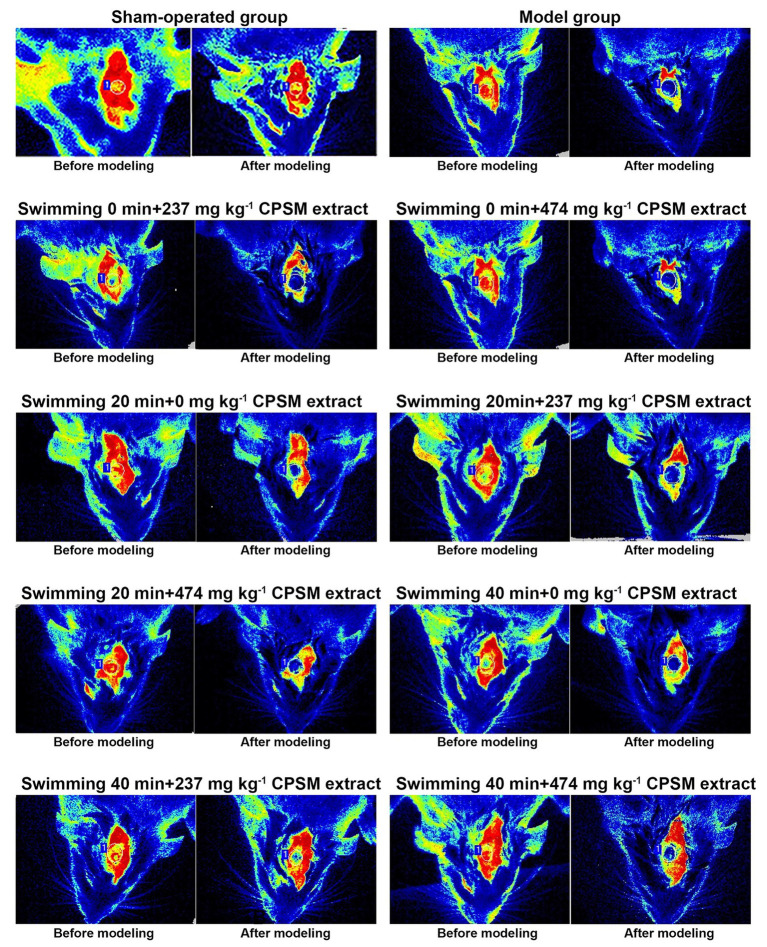
Blood perfusion changes before and after cerebral thrombosis in rats.

Firstly, the combination of CPSM extract and swimming suppressed the changes in blood perfusion (Figure 2). A significant interaction effects between CPSM extract and swimming was found (*F* = 3.36, *p* < 0.05). Further analysis of the separate effects of swimming and CPSM showed that when the CPSM extract dose was fixed at low, the separate effects of swimming (*F* = 10.79, *p* < 0.001) had a significant difference. When the swimming time was fixed at 40 min, there is a significant difference on the separate effects of CPSM extract (*F* = 4.61, *p* < 0.05). Continuing the pairwise comparison of individual effects, and it was found that the combination of 237 mg kg^−1^, CPSM extract and 40 min swimming remarkably reduced the difference in blood perfusion before and after modeling (ranging from −137.72 ± 34.39 PU to −78.47 ± 23.27 PU, *p* < 0.05), which suggested that the above-mentioned combination might be the optimum and was important in increasing blood perfusion, as shown in Table 1.

**Table 1:** Blood perfusion changes, infarct area proportion, and vascular injury area proportion in different treatment groups (mean ± SD)^a^.

Swimming (min)	CPSM (mg kg^−1^)	Blood perfusion changes (PU)		Infarct area proportion (%)		Vascular injury area proportion (%)	
Sham-operated		−24.65 ± 5.87		0		0	
0	0	−137.72 ± 34.39		11.90 ± 3.05		27.65 ± 8.92	
0	237	−146.10 ± 30.38		10.90 ± 1.86		23.35 ± 6.25	
0	474	−119.81 ± 30.40		10.13 ± 3.06		22.05 ± 4.78	
20	0	−119.86 ± 29.95		10.62 ± 3.18		20.92 ± 6.59	
20	237	−116.98 ± 29.27		8.32 ± 2.80		15.21 ± 3.67	
20	474	−112.65 ± 31.49		10.28 ± 2.85		21.58 ± 6.32	
40	0	−118.70 ± 21.68		4.76 ± 1.04		20.54 ± 6.49	
40	237	−78.47 ± 23.27		7.45 ± 1.43		11.54 ± 1.33	
40	474	−127.28 ± 44.34		4.89 ± 1.47		18.15 ± 6.23	
Two-way ANOVA		*F*	*p*	*F*	*p*	*F*	*p*
Swimming		4.86	0.01	21.73	<0.001	7.87	<0.001
CPSM		0.31	0.73	0.39	0.68	6.70	<0.001
CPSM × swimming		3.36	0.02	0.55	0.70	1.67	0.17

a All data are expressed as the mean ± SD. The GLM procedure was applied for a two-way ANOVA to test for synergy. Comparisons between each pair of factor levels were analyzed using a Bonferroni *post hoc* test. A value of *p* < 0.05 was considered statistically significant.

**Figure 3:**
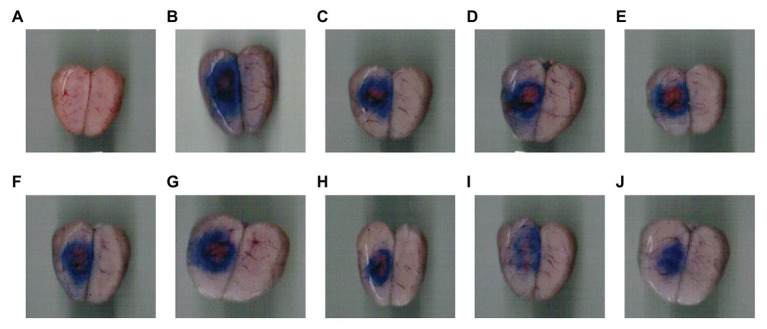
Cerebral infarction area and vascular injury area in rats with focal cerebral thrombosis. **(A)** Sham-operated group, **(B)** Model group, **(C)** Swimming 0 min + 237 mg kg^−1^CPSM extract, **(D)** Swimming 0 min + 474 mg kg^−1^CPSM extract, **(E)** Swimming 20 min + 0 mg kg^−1^CPSM extract, **(F)** Swimming 20 min + 237 mg kg^−1^CPSM extract, **(G)** Swimming 20 min + 474 mg kg^−1^CPSM extract, **(H)** Swimming 40 min + 0 mg kg^−1^CPSM extract, **(I)** Swimming 40 min + 237 mg kg^−1^CPSM extract, and **(J)** Swimming 40 min + 474 mg kg^−1^CPSM extract.

Secondly, for the proportion of cerebral infarction area and the proportion of vascular injury area (Figure 3), the interaction between CPSM extract and swimming did not occur (*F* = 0.55, *p* = 0.69; *F* = 1.67, *p* = 0.17). However, swimming for different times, as a single factor, displayed a significant effect on decreasing the proportion of cerebral infarction area and the proportion of vascular injury area in rats (*F* = 21.73, *p* < 0.001; *F* = 7.86, *p* < 0.01), as shown in Table 1.

### Detection of Endothelial Factors

#### Comparison of the Sham-operated Group and the Model Group

In the model rats, the serum levels of PAI-1 (ranging from 34769.83 ± 1323.89 pg ml^−1^ to 64492.85 ± 6468.98 pg ml^−1^, *p* < 0.05), ET-1 (ranging from 65.79 ± 7.12 pg ml^−1^ to 166.96 ± 22.15 pg ml^−1^, *p* < 0.05), andTXB_2_ (ranging from 64.17 ± 7.15 pg ml^−1^ to 158.27 ± 21.50 pg ml^−1^, *p* < 0.05) were significantly higher than those in the sham-operated rats. Meanwhile, compared with the sham-operated group, the t-PA (ranging from 11542.91 ± 1532.31 pg ml^−1^ to 5829.23 ± 838.39 pg ml^−1^, *p* < 0.05) and 6-keto-PGFIα (ranging from 147.14 ± 13.98 pg ml^−1^ to 57.75 ± 6.32 pg ml^−1^, *p* < 0.05) values were evidently decreased in the model group (Figure 1C).

#### The Combination of CPSM Extract and Swimming

As shown in Table 2, apparent interaction effects were found between CPSM extract and swimming on increasing the t-PA levels (*F* = 2.79, *p* < 0.05) and 6-keto-PGF1α (*F* = 19.98, *p* < 0.001), while decreasing PAI-1 (*F* = 4.74, *p* < 0.001) and TXB_2_ (*F* = 5.37, *p* < 0.001) secretion.

1. For the serum levels of t-PA, the individual effects of swimming and CPSM extract were further analyzed. The results showed that: when the CPSM extract was fixed at 0, low, and high doses, the individual effects of swimming were significantly different (*F* = 49.59, *p* < 0.001; *F* = 14.27, *p* < 0.001; and *F* = 20.51, *p* < 0.001). When the swimming time was fixed at 0, 20, and 40 min, the individual effects of the CPSM extract had significant differences (*F* = 42.26, *p* < 0.001; *F* = 17.80, *p* < 0.001; and *F* = 13.33, *p* < 0.001). Continuing to compare the individual effects in pairs and it was found that the 474 mg kg^−1^, CPSM extract combined with 40 min swimming exhibited a notable increasing effect (ranging from 5829.23 ± 838.39 pg ml^−1^ to 11042.35 ± 587.88 pg ml^−1^, *p* < 0.05).
2. For the serum levels of PAI-1, the individual effects of swimming and CPSM extract were further analyzed. The results showed that: when the CPSM extract was fixed at 0, low, and high doses, the individual effects of swimming were significantly different (*F* = 38.59, *p* < 0.001; *F* = 9.39, p < 0.001; and *F* = 5.73, *p* < 0.01). When the swimming time was fixed at 0, 20, and 40 min, the individual effects of the CPSM extract had significantly different (*F* = 67.97, *p* < 0.001; *F* = 17.70, *p* < 0.001; and *F* = 18.34, *p* < 0.001). Continuing to compare the individual effects in pairs and it was found that the 474 mg kg^−1^ CPSM extract combined with 40 min swimming had a remarkably decreased PAI-1 secretion (ranging from 64492.85 ± 6468.98 pg ml^−1^ to 38285.89 ± 2814.35 pg ml^−1^, *p* < 0.001).
3. For the serum levels of 6-keto-PGF1α, further analysis of the individual effects of swimming and CPSM extract showed that: when the CPSM extract was fixed at 0, low, and high doses, the individual effects of swimming were significantly different (*F* = 211.95, *p* < 0.001; *F* = 45.36, *p* < 0.001; and *F* = 50.48, *p* < 0.001). When the swimming time was fixed at 0, 20, and 40 min, the individual effects of the CPSM extract were significantly different (*F* = 26.24, *p* < 0.001; *F* = 14.80, *p* < 0.001; and *F* = 7.11, *p* < 0.01).
4. For the serum levels of TXB_2_, further analysis of the individual effects of swimming and CPSM extract, and the results showed as follows: when the CPSM extract was fixed at 0, low, and high doses, the individual effects of swimming were significantly different (*F* = 3.91, *p* = 0.026; *F* = 31.99, *p* < 0.001; and *F* = 32.58, *p* < 0.001), when the swimming time was fixed at 0, 20, and 40 min, the individual effects of the CPSM extract were all significantly different (*F* = 7.89, *p* < 0.001; *F* = 15.55, *p* < 0.001; and *F* = 46.03, *p* < 0.001). Continuing to compare the individual effects in pairs and it was found that the group composed of 474 mg kg^–1^, CPSM extract and 40 min swimming had significantly decreased TXB_2_ secretion (ranging from 158.27 ± 21.50 pg ml^−1^ to 82.15 ± 12.41 pg ml^−1^, *p* < 0.05).

**Table 2:** Endothelial function factors in different treatment groups (mean ± SD)^a^.

Swimming (min)	CPSM (mg kg^−1^)	t-PA (pg ml^−1^)		PAI-1 (pg ml^−1^)		ET-1 (pg ml^−1^)		6-Keto-PGFI_α_ (pg ml^−1^)		TXB_2_ (pg ml^−1^)	
Sham-operated		11542.91 ± 1532.31		34769.83 ± 1323.89		65.79 ± 7.12		147.14 ± 13.98		64.17 ± 7.15	
0	0	5829.23 ± 838.39		64492.85 ± 6468.98		166.96 ± 22.15		57.75 ± 6.32		158.27 ± 21.50	
0	237	8223.36 ± 908.86		48827.80 ± 4452.26		134.53 ± 6.19		81.47 ± 9.03		142.68 ± 11.46	
0	474	8769.49 ± 637.01		44455.57 ± 2621.27		129.64 ± 6.55		88.44 ± 8.86		133.88 ± 10.80	
20	0	7874.40 ± 1076.80		53255.25 ± 3832.51		143.47 ± 6.41		129.04 ± 8.02		149.53 ± 9.18	
20	237	9455.80 ± 437.15		44569.45 ± 928.96		128.47 ± 7.16		105.12 ± 9.70		133.68 ± 12.58	
20	474	9884.82 ± 219.63		42775.73 ± 1818.98		112.54 ± 13.72		123.58 ± 14.04		113.59 ± 5.23	
40	0	9210.72 ± 271.99		49138.89 ± 1532.58		138.18 ± 6.99		142.87 ± 9.60		140.79 ± 8.49	
40	237	10087.91 ± 435.67		40662.67 ± 3043.49		100.08 ± 10.51		125.53 ± 5.19		94.12 ± 7.44	
40	474	11042.35 ± 587.88		38285.89 ± 2814.35		90.46 ± 11.61		132.41 ± 3.60		82.15 ± 12.41	
Two-way ANOVA		*F*	*p*	*F*	*p*	*F*	*p*	*F*	*p*	*F*	*p*
Swimming		77.30	<0.001	42.70	<0.001	46.68	<0.001	259.43	<0.001	58.66	<0.001
CPSM		66.34	<0.001	92.03	<0.001	64.62	<0.001	8.19	<0.001	59.87	<0.001
CPSM × swimming		2.79	0.04	4.74	<0.001	2.15	0.09	19.98	<0.001	5.37	<0.001

a All data are expressed as the mean ± SD. The GLM procedure was applied for a two-way ANOVA to test for synergy. Comparisons between each pair of factor levels were analyzed using a Bonferroni *post hoc* test. A value of *p* < 0.05 was considered statistically significant.

In the end, for the serum levels of ET-1, the interaction between CPSM extract and swimming did not occur (*F* = 2.15, *p* = 0.09). However, swimming for different times (*F* = 46.68, *p* < 0.001) and CPSM extract with different dosages (*F* = 64.62, *p* < 0.001), both as a single factor, had an evident influence on decreasing ET-1 secretion.

In brief, the combination of CPSM extract with swimming could increase the level of serum t-PA with a decreased PAI-1 secretion and the increased 6-keto-PGF1α levels and decreased the secretion of TXB_2_ was likely found. The above-mentioned results might suggest that the combined treatment of CPSM extract and swimming adjusted the balance of t-PA/PAI-1 and 6-keto-PGF1α/TXB_2_, which might improve the endothelial functions in focal cerebral ischemia. Therefore, the combined treatment of CPSM extract and swimming might inhibit thrombus formation by regulating the stability of the fibrinolytic system as well as the balance of 6-keto-PGF1α/TXB_2_.

### Detection of vWF, Adhesion Molecules VCAM-1, and ICAM-1 Expression in Rats With Focal Cerebral Ischemia

**Figure 4:**
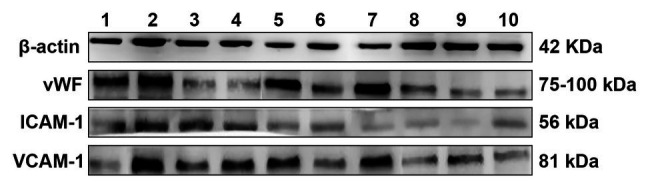
Von Willebrand factor (vWF), vascular cell adhesion molecule-1 (VCAM-1), and intercellular adhesion molecule-1 (ICAM-1) protein expression in rat cerebral infarction tissue after photochemical induction **(1)** Sham-operated group, **(2)** Model group, **(3)** Swimming 0 min + 237 mg kg^−1^CPSM extract, **(4)** Swimming 0 min + 474 mg kg^−1^CPSM extract, **(5)** Swimming 20 min + 0 mg kg^−1^CPSM extract, **(6)** Swimming 20 min + 237 mg kg^−1^CPSM extract, **(7)** Swimming 20 min + 474 mg kg^−1^CPSM extract, **(8)** Swimming 40 min + 0 mg kg^−1^CPSM extract, **(9)** Swimming 40 min + 237 mg kg^−1^CPSM extract, and **(10)** Swimming 40 min + 474 mg kg^−1^CPSM extract.

The western blot protein expression results are shown in Figure 4. Firstly, compared with the sham-operated group, protein investigation revealed that the expression levels of vWF (ranging from 0.11 ± 0.03 to 0.43 ± 0.08), VCAM-1 (ranging from 0.21 ± 0.04 to 0.83 ± 0.26, *p* < 0.05), and ICAM-1 (ranging from 0.15 ± 0.05 to 0.67 ± 0.22, *p* < 0.05) in the model rats were significantly increased (*p* < 0.05), as shown in Figure 1D.

Moreover, significant interactive effects were found between CPSM extract and swimming on decreasing the vWF (*F* = 9.29, *p* < 0.001), VCAM-1 (*F* = 8.59, *p* < 0.001), and ICAM-1 (*F* = 3.62, *p* < 0.01) protein expression, as shown in Table 3. (I) For the protein expression of vWF, further analysis of the individual effects of swimming and CPSM extract showed that: when the CPSM extract was fixed at low and high doses, the individual effects of swimming were significantly different (*F* = 9.24, *p* < 0.001; *F* = 32.01, *p* < 0.001), when the swimming time was fixed at 20 and 40 min, the individual effects of the CPSM extract were all significantly different (*F* = 15.36, *p* < 0.001; *F* = 6.44, *p* < 0.001;). Further pairwise comparison of the individual effects signified that the combined treatment of 474 mg kg^−1^ CPSM extract with 40 min swimming exhibited a marked effect on reducing vWF expression (ranging from 0.43 ± 0.08 to 0.15 ± 0.02, *p* < 0.05). (II) For the protein expression of VCAM-1, further analysis of the individual effects of swimming and CPSM extract showed that: when the CPSM extract was fixed at 0 and high doses, the individual effects of swimming were significantly different (*F* = 15.34, *p* < 0.001; *F* = 5.92, *p* < 0.01; *p* < 0.05). When the swimming time was fixed at 20 min, the individual effects of the CPSM extract were significantly different (*F* = 4.93, *p* < 0.05). (III) For the protein expression of ICAM-1, further analysis of the individual effects of swimming and CPSM extract showed that: when the CPSM extract was fixed at 0, low, and high doses, there were significant differences in the individual effects of swimming (*F* = 20.34, *p* < 0.001; *F* = 12.96, *p* < 0.001; and *F* = 10.53, *p* < 0.001). When the swimming time was fixed at 0 and 20 min, the individual effects of the CPSM extract were all significantly different (*F* = 7.47, *p* < 0.01; *F* = 7.95, *p* = 0.001).

**Table 3:** vWF, VCAM-1, and ICAM-1 protein expression in different treatment groups (mean ± SD)^a^.

Swimming (min)	CPSM (mg kg^−1^)	vWF		VCAM-1		ICAM-1	
Sham-operated		0.11 ± 0.03		0.21 ± 0.04		0.15 ± 0.05	
0	0	0.43 ± 0.08		0.83 ± 0.26		0.67 ± 0.22	
0	237	0.42 ± 0.13		0.64 ± 0.17		0.40 ± 0.12	
0	474	0.53 ± 0.14		0.72 ± 0.22		0.38 ± 0.13	
20	0	0.29 ± 0.05		0.45 ± 0.15		0.38 ± 0.12	
20	237	0.34 ± 0.09		0.78 ± 0.23		0.63 ± 0.20	
20	474	0.60 ± 0.17		0.63 ± 0.21		0.69 ± 0.19	
40	0	0.34 ± 0.09		0.26 ± 0.08		0.14 ± 0.04	
40	237	0.16 ± 0.03		0.519 ± 0.16		0.22 ± 0.07	
40	474	0.15 ± 0.02		0.37 ± 0.10		0.35 ± 0.04	
Two-way ANOVA		*F*	*p*	*F*	*p*	*F*	*p*
Swimming		24.25	<0.001	7.87	<0.001	17.08	<0.001
CPSM		5.82	0.01	6.70	<0.001	2.39	0.10
CPSM × swimming		9.29	<0.001	8.59	<0.001	3.62	0.01

Expression of vWF, VCAM-1, and ICAM-1were normalized with respect to β-actin.

a All data are expressed as the mean ± SD. The GLM procedure was applied for a two-way ANOVA to test for synergy. Comparisons between each pair of factor levels were analyzed using a Bonferroni *post hoc* test. A value of *p* < 0.05 was considered statistically significant.

In brief, these results demonstrated that the combined treatment of CPSM extract and swimming could inhibit the protein vWF, VCAM-1, and ICAM-1 expression and might protect ECs function and reduce inflammatory response to inhibit thrombosis.

### Expression of t-PA, PAI-1, and ICAM-1 Genes

The relative expression levels of t-PA, PAI-1, and ICAM-1 genes were quantified by the 2^−ΔΔCt^ method. Firstly, without the intervention of shear stress and drugs, established a model of TNF-α injured HUVECs, compared with the control group, the relative expression of t-PA mRNA (ranging from 2.60 ± 0.23 to 1.02 ± 0.16, *p* < 0.05) in the model group was significantly downregulated, while the relative expression of PAI-1 mRNA (ranging from 0.62 ± 0.20 to 1.83 ± 0.24, *p* < 0.05) and ICAM-1 mRNA (ranging from 0.69 ± 0.13 to 1.81 ± 0.36, *p* < 0.05) was significantly upregulated, as shown in Figure 1E, indicating that the preparation of this model was successful.

However, as shown in Table 4, no significant interactive effect was found between CPSM extract and shear stress on t-PA mRNA expression. In fact, CPSM extract and shear stress, as a single factor, could upregulate the expression of t-PA mRNA (*p* < 0.05). Moreover, the t-PA mRNA upregulation by fluid shear stress (0.9 Pa) was more effective than the action of low shear stress (0.1 Pa) at the same doses of CPSM extract, whereas at the same shear stress level, 300 μg ml^−1^ CPSM extract significantly promoted the t-PA mRNA expression. In addition, no significant interactive effect was found between CPSM extract and shear stress on the expression of PAI-1 mRNA in TNF-α-stimulated HUVECs (*F* = 0.45, *p* = 0.65). As a single factor, the main effect of shear stress was not significant, while the main effect of CPSM extract (*p* < 0.01) behaved remarkably significant. Under the condition of 0.9 Pa shear stress, 150 and 300 μg ml^−1^ CPSM extract downregulated the PAI-1 mRNA expression (*p* < 0.01). Noteworthy, interactive effects were observed between CPSM extract and shear stress on ICAM-1 mRNA expression (*F* = 6.858, *p* < 0.01). Further analysis of the shear stress and the individual effect of the CPSM extracts showed that when the shear stress was fixed at 0.1 and 0.9 Pa, the difference in the individual effect of the CPSM extract was statistically significant (*F* = 12.49, *p* = 0.01; *F* = 21.18, *p* < 0.001), when the CPSM extract was fixed at 150 μg·ml^−1^, the difference in the individual effects of shear stress was statistically significant (*F* = 9.41, *p* = 0.01). Continuing to compare the individual effects, the results signified that the combination of 0.9 Pa shear stress and 150 μg·ml^−1^ CPSM extract downregulated ICAM-1 mRNA expression was the most significant (ranging from 1.81 ± 0.31to 0.99 ± 0.12, *p* < 0.05).

**Table 4:** Relative expression levels of tissue plasminogen activator (t-PA) mRNA, plasminogen activator inhibitor type-1 (PAI-1) mRNA, and ICAM-1 mRNA in human umbilical vein endothelial cells (HUVECs) stimulated by TNF-α in different treatment groups (mean ± SD)^a^.

Shear stress (Pa)	CPSM (μg·ml^−1^)	t-PA mRNA		PAI-1 mRNA		ICAM-1 mRNA	
Control group		2.60 ± 0.23		0.62 ± 0.20		0.69 ± 0.13	
Model group		1.03 ± 0.16		1.84 ± 0.24		1.81 ± 0.36	
0.1	0	0.83 ± 0.22		1.76 ± 0.55		1.81 ± 0.31	
0.1	150	1.01 ± 0.20		1.15 ± 0.21		1.59 ± 0.31	
0.1	300	1.34 ± 0.24		0.67 ± 0.10		0.89 ± 0.21	
0.9	0	0.93 ± 0.16		1.55 ± 0.13		2.14 ± 0.12	
0.9	150	1.13 ± 0.22		0.93 ± 0.20		0.99 ± 0.12	
0.9	300	2.34 ± 0.58		0.71 ± 0.15		1.11 ± 0.26	
Two-way ANOVA		*F*	*p*	*F*	*p*	*F*	*p*
Shear stress		5.72	0.03	0.95	0.35	0.01	0.92
CPSM		11.57	0.01	19.16	< 0.001	26.82	< 0.001
CPSM × shear stress		3.01	0.08	0.45	0.65	6.858	0.01

Relative expression levels of t-PA mRNA, PAI-1mRNA, and ICAM-1 mRNA in HUVECs were calculated using 2−^ΔΔCt^ method from triplicate preformation.

a All data are expressed as the mean ± SD. The GLM procedure was applied for a two-way ANOVA to test for synergy. Comparisons between each pair of factor levels were analyzed using a Bonferroni *post hoc* test. A value of *p* < 0.05 was considered statistically significant.

## Discussion

Cerebral thrombosis was first described in 1825 by Ribes, who reported a 45-year-old man that presented severe headache for 6 months, epilepsy, and delirium (Yapijakis, 2017). Cerebrovascular events have become the major killer of people’s life all over the world. Based on previous studies, activated platelets play a pivotal role in the formation of pathogenic thrombi. Oral antiplatelet drugs are a milestone in the therapy of cardiovascular and cerebrovascular atherothrombotic diseases and provide the primary and secondary prevention strategy to combat such diseases (Liu et al., 2012). However, the effectiveness, security, and prolonging the use of oral antiplatelet drugs have garnered particular attention in recent years. Despite their proven benefit, antiplatelet resistance and numerous adverse reactions, including serious bleeding risk (digestive and nervous systems); still occur in those taking antiplatelet drugs (Juurlink et al., 2009). Modern medicine and pharmacology have conducted considerable exploration, and latest agents and treatment strategies are developed in recent years.

### Role of Shear Stress in the Procession of Thrombosis in Rats

Shear stress induced by blood flow plays a critical role in the development of thrombosis. Thrombus formation is related to changes in shear stress because of stenotic lesions and dilated lesions of arteries. With regard to stenotic lesions, increased shear stress can directly expose and/or activate GPIIb/IIIa receptors, whose ligand is vWF, and cause platelets to aggregate and form thrombi (O’Brien, 1990). By contrast, shear stress can be reduced because of decreased artery flow velocity and dilation of artery. This reduced shear stress leads to thrombus formation because of thrombogenicity caused by decreased endothelial function, leading to increased platelet aggregation and coagulation and decreased fibrinolysis (Ohkubo et al., 2007). Evidence is accumulating that blood flow-induced shear stress has emerged as an essential feature of focal cerebral ischemia. Many studies had proved that the disturbed flow may be related to cerebral ischemia. The cerebral blood circulation is a low pressure-high flow circuit. Analysis of shear stress and its effects in the cerebral vasculature *in vivo* is still limited. In addition, research has shown that the peak shear stress of pial surface vessels is 39 ± 14 dyn·cm^−1^ (Rovainen et al., 1993). This result reflects flow in penetrating arteries to capillaries only within the surface of the brain. Alternatively, regular physical training on decreasing the thrombotic tendency in rat cerebral vessels has been widely appreciated (Sasaki et al., 1995).

### The Synergistic Effects of Combined CPSM Extract With Swimming in Focal Cerebral Thrombosis in Rats

Although most animal models of infarction have investigated embolic models and their recanalization, few reports have studied the models of thrombosis. RB is an efficient photosensitizer. The use of the photosensitive dye RB to produce a thrombotic lesion in the rat brain is a reproducible model for studying the hemodynamic and metabolic consequences of focal cerebral ischemia (Grome et al., 1988; Jung et al., 2013). Further studies indicated that this focal cerebral ischemia model is based on RB, which causes the production of large amount of oxygen radicals under specific light conditions and damages endothelia of cerebral blood vessels leading to platelet aggregation, thrombus formation, and occlusion of blood vessels (Watson et al., 1986). Cerebral infraction is a complex disease caused by various risk factors, which make finding effective prevention and treatment methods challenging. Modern medical strategy depends on drugs and interventional therapy. In Chinese medicine, promoting blood circulation by removing blood stasis of Chinese herbs is applied clinically to prevent and treat cardiovascular and cerebrovascular diseases. Therefore, we adopted photochemically induced focal cerebral thrombosis in rats and intended to use it to observe the functional recovery after the administration of CPSM extract. In this study, the changes in blood perfusion at the cerebral infarction site were measured before and after the modeling of rats, and there was a significant interaction between swimming and CPSM to inhibit the decrease in blood perfusion at the thrombus site after modeling (*F* = 3.364, *p* = 0.016). This shows that the combined effect of swimming training and CPSM can enhance the blood flow of the lesion and inhibit the decrease of blood perfusion. In other words, the combined strategy might be an effective way to prevent and treat embolization in the setting of thrombosis. CPSM extract and physical training might also have effects on cerebrovascular function that might modify their blood flow. Swimming alone can significantly inhibit the formation of cerebral infarction and inhibit vascular damage in rats (*p* < 0.01), which shows that swimming training can effectively prevent local thrombosis and peripheral blood vessel damage. The results of this study further prove that exercise possibly produce biological effects by regulating the shear stress of blood flow, which it is the biological pharmacological basis of the theory of promoting blood circulation and removing blood stasis.

Moreover, previous studies showed that ECs are highly sensitive to shear stress. ECs might experience a variety of alterations in thrombus formation. t-PA, which is the major intravascular plasminogen activator *in vivo*, is rapidly neutralized by PAI-l. As a risk factor for ischemic cardiovascular events, the elevated PAI-1 promotes thrombus formation and accelerates fibrinolysis. The balance of 6-keto-PGF1α/TXB_2_ has important physiological and pathological significance for controlling hemostatic mechanism and preventing thrombosis. Our results indicated that the joint effect of swimming combined with CPSM extract increased t-PA release (*F* = 2.788, *p* = 0.035) and decreased the level of PAI-1 (*F* = 4.744, *p* = 0.002). The combined effect of swimming and CPSM can increase the secretion of 6-keto-PGF1α (*F* = 19.98, *p* < 0.001) and decrease the level of TXB_2_ (*F* = 5.366, *p* < 0.01). These findings may further illustrate that swimming and CPSM interventions can adjust the balance between t-PA/PAI-1, 6-keto-PGF1α/TXB_2_ to improve endothelial dysfunction caused by abnormal shear stress and maintain the stability of fibrinolytic system. Meanwhile, inhibit vasoconstriction and platelet aggregation, thereby improving the occurrence and development of thrombotic diseases.

Von Willebrand factor is a macromolecular glycoprotein with adhesion function. It is a specific marker that reflects the dysfunction of vascular ECs. It occurs at the stage of platelet adhesion. Meanwhile, it can mediate the adhesion of platelets to damaged vascular ECs and accelerate the combination of vWF, fibrosis protein, and the cell membrane glycoprotein on the surface of platelets to promote the activation of platelets and further induce thrombosis. The ICAM-1 and VCAM-1 are members of the immunoglobulin superfamily of adhesion molecules (Dianzani et al., 2008). Under normal physiological conditions, vascular ECs express almost no other adhesion molecules except for weakly expressing ICAM-1. The vascular endothelium can inhibit the adhesion of leukocytes to it. Investigations indicate that the expression of ICAM-1 was altered after injury in rat thrombosis vessels (Isogai et al., 2004). Some researchers have proven that shear stress can modulate the expression of ICAM-1. Laminar shear stress under physiological conditions has anti-inflammatory and anti-adhesion effects. Acting physiological laminar shear stress on ECs can downregulate the expression of adhesion molecules, while applying low shear stress increases the adhesion of monocytes and ECs and upregulates expression of the adhesion molecule ICAM-1 and VCAM-1.

The results from the present study show that the combined application of swimming and CPSM extract decreased expression, which was characterized by a significant interaction. This indicates that the combined application of quantitative exercise training and CPSM can prevent leukocytes and platelets from adhering to damaged vascular ECs by inhibiting the protein expression of vWF, ICAM-1, and VCAM-1, thereby protecting the vascular endothelium and inhibiting thrombosis. Thereby, delaying the further development of thrombotic diseases.

### The Synergistic Effect of CPSM Extract Combined With Shear Stress on the TNF-α-Induced HUVECs

Low shear stress induced inflammatory responses are normally considered a vital process in the development of cerebral infraction. Song et al. (2001) and Nyska et al. (2003) reported a specific upregulation in ICAM-1 expression on rat brain microvascular ECs with two magnitudes of laminar shear stresses (0.2 and 0.4 dyn/cm^2^). Similar results were found in a study from Tsuboi et al. (1995); they found that the application of shear stress increased the cell surface expression of ICAM-1 2.7 times the control level 4 h after the onset of flow. Walpola et al. (1995) showed that VCAM-1 expression was increased under low shear stress. Nyska et al. (2003) revealed that the appearance of VCAM-1 immunostaining correlated with the development of thrombosis located in the same structures. TNF-α, a major inflammatory cytokine, induces inflammatory responses by enhancing the expression of inflammatory mediators (Kim et al., 2010). In addition, we further studied the effect of CPSM extract combined with shear stress on the TNF-α-induced inflammatory conditions of HUVECs. Hemodynamic forces altered EC functions by changing the expression of genes related to endothelial functions. Gong et al. (2018) indicated that shear stress-dependent genes have strong relationship with thrombosis and modulation of the inflammatory responses (Gong et al., 2018). The present study investigate the expression of genes related to inflammatory cytokines, thrombosis, and dissolution, such as ICAM-1, t-PA, and PAI-1, and the influences of shear stress combined with CPSM. The results suggested that the combination of shear stress and CPSM extract could downregulate the gene expression of ICAM-1 by which endothelial inflammatory response was alleviated.

Cerebral ischemia is a multifactorial pathology involving different cerebral cellular components. Modern medicines are undoubtedly effective, but they are also costly and risky. Therefore, alternative therapies and novel antithrombotic agents should be developed, and natural products from TCM are increasingly explored. CPSM extract is a combination of extracts from the fruit of CP and the root of SM and is utilized in the management of thrombotic diseases. The safety and efficacy of CPSM have been popularly accepted in China. Exercise training can protect against thrombotic disorders, and the underlying mechanisms of this effect may be related to the suppression of inflammatory responses and improvement of endothelial dysfunction.

The results presented in this report are important and encouraging, because the prevention and treatment of focal cerebral infraction can be achieved from exercise in combination with CPSM extract, rather than an intervention of synthetic drugs. However, further analysis should be conducted to investigate the mechanisms induced by the combination of exercise and medicines.

### Concluding Remarks and Study Limitation

Biomechanopharmacology (Liao et al., 2006), as a new borderline discipline, mainly advocates the joint efficiency of biomechanical and pharmacological interventions in the prevention and treatment of cardio-cerebral vascular diseases. On the one hand, much evidence indicate that exercise training may exert some actions on inflammation, endothelial dysfunction, thrombosis, and lipoprotein retention, so the benefits of exercise for achieving joint biomechanical and pharmacological effects have been widely recognized. However, on the other hand, there still exist some limitations in experiment methods, though we have been trying to find approaches to solution. For example, when rats swim, water is not a suitable environment, swimming training may make them feel stressed. Therefore, in future studies, it should be considered that the non-swimming group of rats will be placed in shallow water to eliminate the effect of stress as much as possible. In addition, this experiment investigated the secretion of ET-1 in rat ECs and did not find a significant interaction between swimming and CPSM, but swimming as a single factor has a significant impact on them, indicating that quantitative exercise training may be possible by inhibiting the secretion of ET-1, it protects EC function. For this result, further research can be conducted.

In conclusion, this study shows that focal cerebral infraction can be prevented by the combination of CPSM (a medicinal factor) and swimming (a physical exercise factor), which interacting on the regulation of vascular endothelial functions and inflammatory factors. The mechanism may be related to regulating the protein expression of vWF, VCAM-1, and ICAM-1, and downregulating the gene expression of ICAM-1. If these effects of the combination of CPSM and swimming are proven in clinical trials, it might be a promising avenue for focal cerebral infraction treatment.

## Data Availability Statement

The raw data supporting the conclusions of this article will be made available by the authors, without undue reservation.

## Ethics Statement

The animal study was reviewed and approved by the Laboratory Animal Ethics Committee of the Institute of Chinese Material Medical, China Academy of Chinese Medical Sciences (Beijing, China). All animal experiments were carried out in accordance with the guidelines and regulations for the care and use of laboratory animal of Center for Laboratory Animal Care, China Academy of Chinese Medical Science.

## Author Contributions

SD, FL, and RL contributed to design the experiments in the study. SD, XY, LW, WW, and LG took part in the experiments. SD, WW, LG, FL, and RL contributed to analyze the data and revise the manuscript. SD, XY, and LW helped to carry out the analysis with constructive discussions. All authors contributed to the article and approved the submitted version.

### Conflict of Interest

The authors declare that the research was conducted in the absence of any commercial or financial relationships that could be construed as a potential conflict of interest.
